# Development of Organosilicon-Based Superhydrophobic Coatings through Atmospheric Pressure Plasma Polymerization of HMDSO in Nitrogen Plasma

**DOI:** 10.3390/ma12020219

**Published:** 2019-01-10

**Authors:** Siavash Asadollahi, Jacopo Profili, Masoud Farzaneh, Luc Stafford

**Affiliations:** 1 Canada Research Chair on Engineering of Power Network Atmospheric Icing (INGIVRE), Université du Québec à Chicoutimi, Saguenay, QC G7H 2B1, Canada; siavash.asadollahi@gmail.com (S.A.); MasoudFarzaneh@uqac.ca (M.F.); 2 Département de Physique, Université de Montréal, Montréal, QC H3T 1J4, Canada; profili.jacopo@gmail.com

**Keywords:** atmospheric pressure plasma jets, plasma polymerization, superhydrophobicity, wetting

## Abstract

Water-repellent surfaces, often referred to as superhydrophobic surfaces, have found numerous potential applications in several industries. However, the synthesis of stable superhydrophobic surfaces through economical and practical processes remains a challenge. In the present work, we report on the development of an organosilicon-based superhydrophobic coating using an atmospheric-pressure plasma jet with an emphasis on precursor fragmentation dynamics as a function of power and precursor flow rate. The plasma jet is initially modified with a quartz tube to limit the diffusion of oxygen from the ambient air into the discharge zone. Then, superhydrophobic coatings are developed on a pre-treated microporous aluminum-6061 substrate through plasma polymerization of HMDSO in the confined atmospheric pressure plasma jet operating in nitrogen plasma. All surfaces presented here are superhydrophobic with a static contact angle higher than 150° and contact angle hysteresis lower than 6°. It is shown that increasing the plasma power leads to a higher oxide content in the coating, which can be correlated to higher precursor fragmentation, thus reducing the hydrophobic behavior of the surface. Furthermore, increasing the precursor flow rate led to higher deposition and lower precursor fragmentation, leading to a more organic coating compared to other cases.

## 1. Introduction

A superhydrophobic surface is defined as a surface for which the equilibrium water contact angle (WCA) is higher than 150° [1] and contact angle hysteresis is lower than 10° [2]. The concept of superhydrophobicity initially emerged from the investigation of natural surfaces with high contact angle and low contact angle hysteresis, notably the lotus leaf (Nelumbo) surface [3]. The superhydrophobic characteristics of the micro-nanostructured and wax coated surface of the lotus leaf was first studied by Dettre and Johnson in 1963 [4]. Since then, several other examples of natural superhydrophobic surfaces have been identified [5]. During the past few decades, many studies have been done trying to mimic some of the structures observed on natural superhydrophobic leaves to develop artificial superhydrophobic surfaces [3,6]. Such surfaces may find a wide range of applications from textile industry to power network design and maintenance [7]. Many studies have been done on water-repellent self-cleaning fabrics [8,9]. Superhydrophobic surfaces may be used in biomedical applications, vessel replacements or wound management [2,10,11]. Since icephobicity (i.e., low adhesion force between ice and the substrate) shows a correlation with superhydrophobicity [12], superhydrophobic coatings can be considered as suitable candidates to reduce the ice accumulation on various structures, notably power network equipment [13,14,15]. Construction industry can benefit from the development of superhydrophobic surfaces for manufacturing self-cleaning windshields and windows [16,17,18]. In marine industry, superhydrophobic coatings can be used to develop anti-fouling surfaces [19] or to assist in oil-water separation [20]. Superamphiphobic surfaces in particular (surfaces with both superhydrophobic and superoleophobic characteristics) may be used for such applications [21]. Furthermore, due to their potential in minimizing the liquid/surface contact area, hydrophobic and superhydrophobic surfaces can be used in anticorrosion application [6,22,23,24].

One of the most promising approaches to surface modification and coating deposition are plasma-based surface treatment methods, due to their high controllability, relatively low cost, low pollution levels, and short treatment times. Such treatments typically involve removing molecules from the surface (plasma etching/sputtering) and/or depositing a different material on the surface (plasma polymerization) using high-energy plasma-generated-species [25]. During the past few decades, plasma-based processes have been used for a wide range of applications, such as deposition of various functional coatings [10,25,26], modifying surface topography and micro/nano texturing [27], treatment of tumors and infectious wounds [28,29,30], or even treatment of food products [31,32]. Depending on the gas pressure in which the plasma is generated, plasma treatment may be carried out in low-pressure or atmospheric-pressure. Low-pressure plasma treatment typically results in more uniform films and may be used for 2D or 3D treatment due to the spatial homogeneity of the reactive species. Atmospheric-pressure plasmas, on the other hand, are easier to generate and maintain since they do not require cost-intensive vacuum pumps and chambers [33]. However, due to the open-air configuration in atmospheric-pressure treatment, plasma treatment of oxidation-sensitive materials becomes limited.

In this study, the development of an organosilicon-based superhydrophobic surface through atmospheric-pressure plasma deposition of hexamethyldisiloxane (HMDSO) is reported. In this specific paper, emphasis is placed on the precursor fragmentation dynamics and the effects of ‘available energy per precursor molecule’ on coating properties. Molecular fragmentation of HMDSO in plasma leads to the deposition of low-surface-energy methyl groups on the substrate, thus reducing the wettability of the surface. It is often argued that the wetting characteristics is directly linked to the degree of precursor fragmentation since the energy required to break Si-C bond (318 kJ/mol) is less than the energy required to break Si-O bond (452 kJ/mol). Therefore, higher plasma energies typically lead to more polar oxide functions on the surface, which will in turn decrease the surface hydrophobicity [25,34,35]. Using a dielectric barrier discharge operating at atmospheric-pressure, Siliprandi et al. have shown that for low HMDSO concentrations (less than 0.3% in their study), the deposition process strongly depends on precursor presence in the plasma. However, beyond this threshold value the deposition process is mostly controlled by the plasma generation power, indicating a power-deficient regime [34]. In other words, fragmentation can be essentially controlled by adjusting the available energy per precursor molecule; this a well-established concept in low-pressure plasma deposition. Gas phase fragmentation and recombination reactions are also dependent on the residence time of plasma-generated-species. Longer residence times can be linked to higher precursor fragmentation, which correlated to higher oxide content in the case of HMDSO deposition. This has been confirmed by investigating the effects of plasma gas velocity and precursor injection position on surface chemical composition [36]. For more information on the applications of plasma technology in development of superhydrophobic surfaces, see Reference [25,37].

More specifically, this work reports the development of superhydrophobic coatings on pre-treated alumina-based substrates through atmospheric-pressure plasma deposition of HMDSO in the jet of an open-to-air nitrogen plasma produced by rotating arc discharges. The effects of precursor flow rate and generation power on precursor fragmentation in the discharge and thus surface chemical composition is demonstrated. Furthermore, the wetting behaviors of all coatings are studied through static and dynamic water contact angle measurement, and the results are correlated with the precursor fragmentation dynamics, surface chemical composition, and surface morphology.

## 2. Experimental Procedure

**Figure 1:**
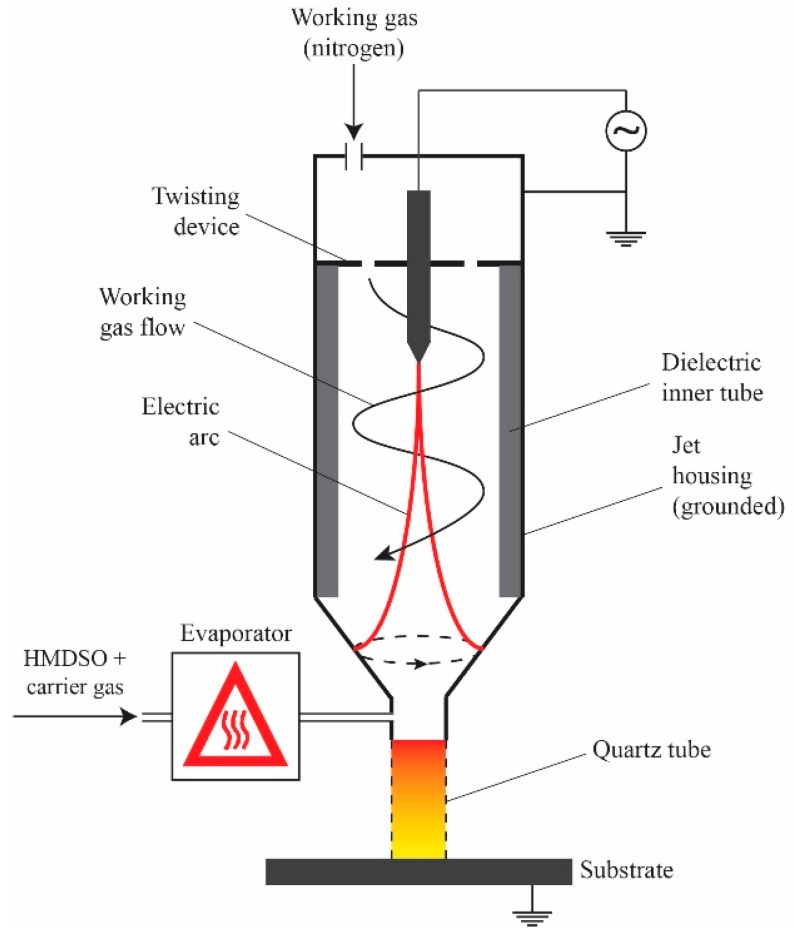
Schematics of the modified APPJ used in this study.

**Figure 2:**
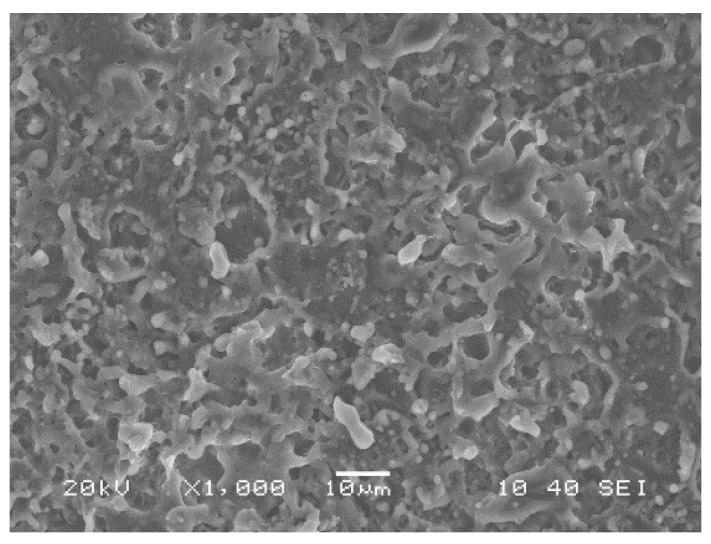
Surface structure of the aluminum surface after pre-treatment [38]. This structure is used as the substrate for deposition of organosilicon based coatings.

30 mm × 50 mm × 1.8 mm samples are cut from Al-6061 sheets provided by ALCAN. For all plasma treatment procedures, a commercial OpenAir AS400 atmospheric pressure plasma jet manufactured by PlasmaTreat^®^ (PlasmaTreat GmbH, Steinhagen, Germany) with a PFW10 nozzle is used. The schematics of the jet are presented in Figure 1. The process is open to ambient air and is carried out at room temperature and in uncontrolled humidity conditions. At first, the samples are exposed to multiple passes of air plasma treatment at very short jet-to-substrate distances to generate an alumina-based micro-roughened porous surface structure on the aluminum substrate (Figure 2). The details of this process, which is driven by the formation of electric discharges between the rotating arc inside the jet body and the substrate, are previously studied by the authors [38]. These “pre-treated” samples are then cleaned in an ultrasonic bath of ethanol and de-ionized water (15 min each at room temperature) to remove any surface contamination prior to coating deposition. For the deposition step, the plasma jet is slightly modified with a quartz tube mounted on the jet-head. This will be discussed in more detail later. HMDSO (Sigma-Aldrich^®^, St. Louis, MO, USA, >98%) was used as the growth precursor. Nominally pure nitrogen gas is purchased from Praxair and is used as received. All samples studied here are prepared by the injection of HMDSO vapor (vaporized at 125 °C) mixed with a carrier gas (nitrogen) into the flowing afterglow region of a nitrogen plasma on pre-treated aluminum surfaces. In total, 3 samples are studied. Two samples are prepared with two different monomer flow rates: 3 g/h and 5 g/h, referred to as PT3 and PT5 samples, respectively. A third sample, referred to as PT5P75, was prepared with the same conditions as PT5 except for the plasma power, which is increased from 500 W to 750 W. It should be noted that plasma deposition is performed under pulsed plasma conditions (duty cycle of 50%) to prevent excessive precursor fragmentation and to limit powder formation. Plasma parameters used for deposition process are also presented in Table 1.

An LR2-T optical emission spectrometer (Toshiba^®^, Tokyo, Japan) with a spectral resolution of 2 nm is used to analyze plasma properties. This spectrometer can acquire optical emission spectra in the range of 200–1200 nm, and the detector is thermoelectrically cooled to optimize the signal-to-noise ratio. All optical emission spectra presented in this paper are recorded with an exposure time of 200 ms. The fiber was placed close to the sample surface (<1 mm above the surface) at 3 cm from the jet axis. A JSM-7600F scanning electron microscope (SEM) manufactured by JEOL^®^ (Tokyo, Japan) is used to acquire images of coating’s surface structure. To improve image clarity, samples are coated with a nanometric layer of carbon prior to SEM analysis. Presence of various chemical functions on the surface was studied through Fourier transform infrared spectroscopy (FTIR). A Bruker Vertex 70 FTIR system is used in ATR mode with a resolution of 2 cm^−1^. Each one of the FTIR spectra presented in this paper is averaged over 32 consecutive scans on the sample. Further chemical analysis on the chemical composition of the surface and the chemical state of silicon is performed using an X-ray photoelectron spectrometer (XPS) with a non-monochromatic Al (max energy 1486.6 eV) anode manufactured by Staib Instruments^®^ (Langenbach, Germany). Scanning parameters used for the acquisition of survey and high-resolution spectra are presented in Table 2. All analysis on the spectra is performed using CasaXPS software (developed by Casa Software Ltd., Telgnmouth, UK). Charge compensation was performed by fitting a synthetic peak in C 1s envelope and then calibrating the peak position it to 284.5 eV.

All water contact angle measurements are performed using a DSA100 goniometer manufactured by Kruss^®^ (Kruss GmbH, Hamburg, Germany). Static contact angle is measured by placing a 4 µL droplet on the surface and calculating the angle at the 3-phase interface based on Young-Laplace approximation. Static contact angle values presented here are the average of at least 15 measurements on different locations and/or samples. To evaluate the dynamic wetting behavior, at first the needle is placed close to the surface and a 4 µL droplet is deposited on the surface. Then, the volume of this droplet is steadily increased to 13 µL with a rate of 3 µL/min. The contact angle on each side of the droplet is measured 5 times per second using the tangent method. Advancing contact angle can then be defined based on the *contact angle* vs. *time* curve. Similarly, the receding contact angle is defined by reducing the droplet volume back to its initial value and constantly measuring the contact angle of the moving interfaces. For more information on this procedure, see [39].

**Figure 3:**
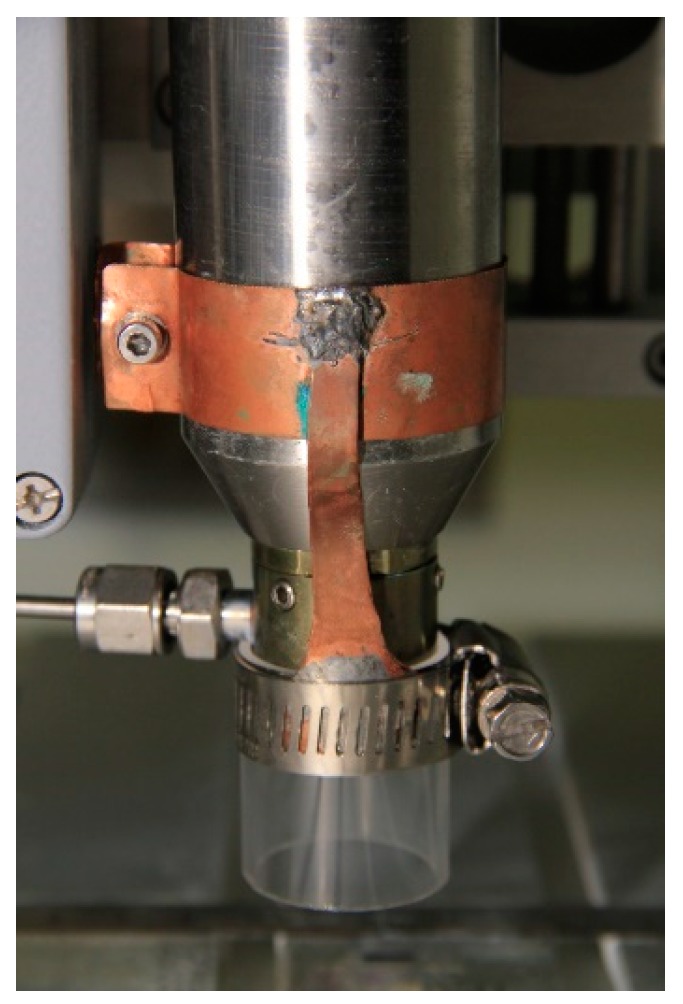
Plasma jet after modification with the quartz tube.

As mentioned before, in the deposition of hydrophobic or superhydrophobic coatings, oxygen incorporation in the coating needs to be limited, particularly in open-to-air atmospheric-pressure plasma conditions. In this context, the plasma jet described above was slightly modified to confine the flowing afterglow region and limit the diffusion of oxygen into the reactive plasma zone. This was achieved by mounting a quartz tube (ID 25 mm, OD 27 mm, and length of 30) on the jet nozzle using a handmade copper clamp (Figure 3). It was immediately observed that confining the plasma jet allows for ignition and maintenance of much weaker plasmas by reducing the loss of ionized species into the ambient atmosphere. This is of significant interest in this work, since lower energy leads to lower precursor fragmentation and thus higher retention of organic functions in the coating structure.

**Figure 4:**
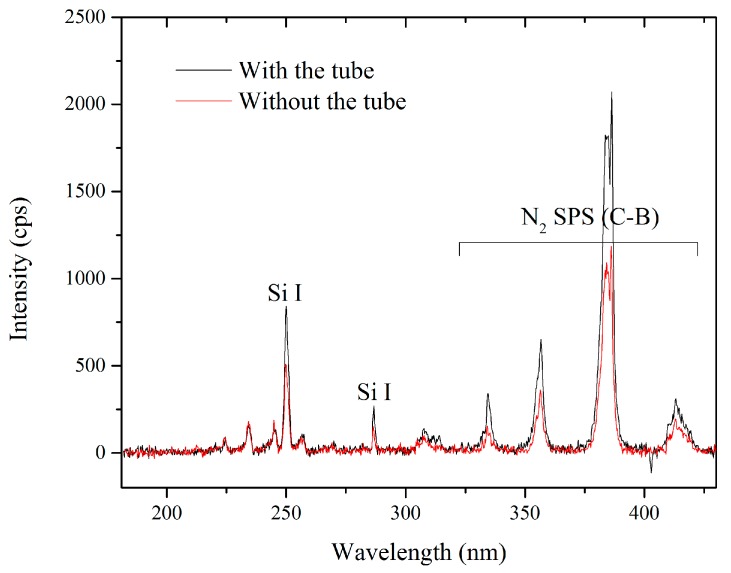
OES spectra acquired from the plasma with and without the quartz tube surrounding the jet-head.

Plasma composition in the presence of the quartz tube was studied using OES and the results are presented in Figure 4. It should be noted that the optical spectra presented here were acquired using a different quartz tube (not pictured here) with an opening at the lowest part. The fiber is placed in front of this opening at a suitable distance (3 cm) so that the optical emission is gathered directly from the discharge zone without interference from the quartz or any possible deposition on the optical fiber. The spectra presented in Figure 4 are acquired with and without the quartz tube surrounding the jet and under PT5 conditions. Only a limited wavelength range (150–450 nm) is presented here, since the emission outside this range was very weak. The spectra are dominated by the nitrogen second positive system, which is typically observed in atmospheric-pressure nitrogen plasmas open to ambient air. Emission intensity is significantly stronger in the spectra acquired with the quartz tube, and since the plasma gas flow rate and the precursor flow rate are identical in both cases, higher intensity is suggestive of a modification of the plasma density and/or the plasma temperatures, at least at the position of OES measurements (<1 mm from sample surface). It is worth mentioning that the presence of Si lines is suggestive of significant precursor fragmentation: atomic Si lines are rarely observed during plasma polymerization in cold, atmospheric-pressure plasmas; this can most likely be attributed to the much higher electron densities and temperatures in the arc-based discharges and flowing afterglow regions than in other systems such as homogeneous dielectric barrier discharges [40]. In fact, Pulpytel et al. have observed atomic Si lines in the optical emission spectrum of air plasma with HMDSO as the precursor in the same reactor operated in comparable experimental conditions [41].

**Figure 5:**
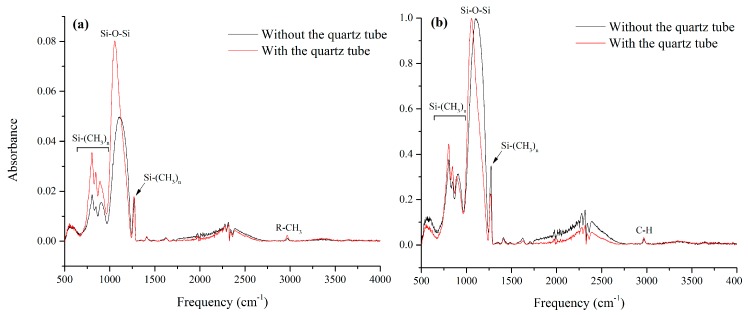
(**a**) Fourier transform infrared spectroscopy (FTIR) spectra of samples prepared with PT5 conditions, with and without the quartz tube surrounding the jet. (**b**) shows the same spectra normalized according to Si-O-Si band.

Chemical composition of the samples acquired from nitrogen plasma deposition on flat aluminum with and without the quartz tube surrounding the jet was studied using FTIR spectroscopy, and the results are presented in Figure 5. The spectral features observed in Figure 5 are similar to those observed in typical organosilicon-based depositions. Comparing the intensity of Si-O-Si band in both cases suggests a lower amount of deposition in the sample prepared without the quartz tube. This can be due to the loss of reactive species due to the jet movement or turbulence in the ambient atmosphere when the jet is not confined. To compare the chemical composition of the coating regardless of the deposition thickness, the fingerprint region of the spectra is also normalized according to the Si-O-Si band and the result is presented as an inset in Figure 5.

The intense band between 1000 cm^−1^ and 1200 cm^−1^ is generally assigned to Si-O-Si and Si-O-C asymmetric stretching modes [42,43]. This band is usually assumed to be the sum of three Gaussian components which correspond to different bond angles in Si-O-Si: TO_2_ mode at 1120 cm^−1^ (170°–180° bond angle), TO_1_ mode at 1070 cm^−1^ (140° bond angle), and TO_3_ mode at 1030 cm^−1^ (120° bond angle) [43]. TO_2_ mode is often associated with fragments of Si-O-Si chains, but in organic films this wavenumber is also populated by Si-O-C stretching mode. TO_1_ is assigned to the in-phase asymmetric stretching vibrational mode of the neighboring SiO_2_ moieties (−O−Si−O−) in a quartz-like structure while TO_3_ mode in SiO_x_C_y_H_z_ films is often observed because of the methyl environment of the bond [44]. The position of Si-O-Si band in the sample prepared with the quartz tube seems to be between the theoretical position of the TO_1_ and TO_3_ mode (max intensity at 1056 cm^−1^).

In comparison, in the absence of the quartz tube, Si-O-Si band is shifted to higher wave numbers (maximum intensity at 1105 cm^−1^), suggesting that without the tube this region is dominated by either Si-O-C bonds or Si-O-Si bond in TO_2_ mode, which may be associated with the fragments of Si-O-Si chains. This, along with the lower deposition in the case without the quartz tube, suggests that the addition of the quartz tube to the plasma jet leads to higher deposition and longer chains of SiO_x_C_y_H_z_ structures.

In both spectra, Si-(CH_3_)_n_ group is easily recognizable by a strong, sharp band at around 1275 cm^−1^ (CH symmetric deformation in Si-CH_3_) together with three bands in the 865–750 cm^−1^ range [45]. This suggests that, even if with the quartz tube the coating seem more inorganic, the Si-O-Si network is at least partially functionalized with methyl groups.

**Figure 6:**
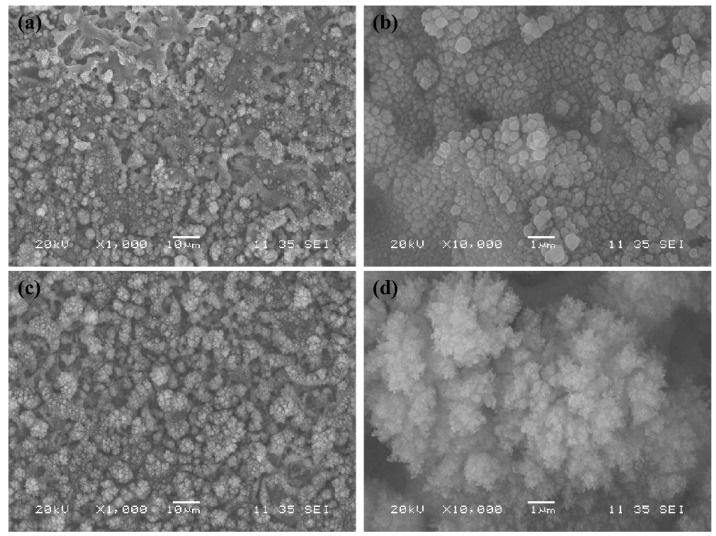
Surface structure of the coatings deposited without a quartz tube surrounding the jet (**a**,**b**) and with the quartz tube (**c**,**d**).

To study the effect of this modification on surface morphology, two samples were prepared under PT5 conditions on the pre-treated aluminum with and without the quartz tube surrounding the jet. Figure 6 compares the surface structure of the coating with and without the quartz tube. Without the quartz tube, the deposition consists of spherical structures covering the micro features obtained after the pre-treatment step (Figure 6a,b). These particles seem to be distributed uniformly on the surface and their diameter range from a few to hundreds of nanometers. On the other hand, surface structure in the presence of the quartz tube (Figure 6c,d) is consisted of dendrite-like structures with multiple levels of roughness, ranging from only a few nanometers to tens of micrometers. Such a hierarchical structure is shown to be ideal for hydrophobic and superhydrophobic applications [46]. Similar features have been reported by Kilicaslan et al. during the deposition of HMDSO-based nanomaterials in atmospheric-pressure plasmas sustained by microwave electromagnetic fields [47]. While spherical silica nanoparticles were obtained in the presence of O_2_ in Ar/HMDSO plasma, dendrite- like SiOCH nanostructures were formed in the absence of O_2_.

In conclusion, it is shown that the addition of a quartz tube around the atmospheric-pressure plasma jet can affect the treatment and deposition dynamics in several ways:

- Facilitates the ignition and maintenance of weaker plasmas, which leads to lower monomer fragmentation and is generally favorable in hydrophobic applications;
- Limits the diffusion of oxygen from the ambient air into the discharge zone;
- Increases the coating thickness by reducing the loss of reactive species into the ambient atmosphere;
- Increases the cross-linking of the silica-like network by increasing the Si-O-Si chain lengths.

All the results presented in the following sections are acquired from the modified jet (i.e., with the quartz tube surrounding the flowing afterglow region).

## 3. Results and Discussion

### 3.1. Optical Emission Spectroscopy

**Figure 7:**
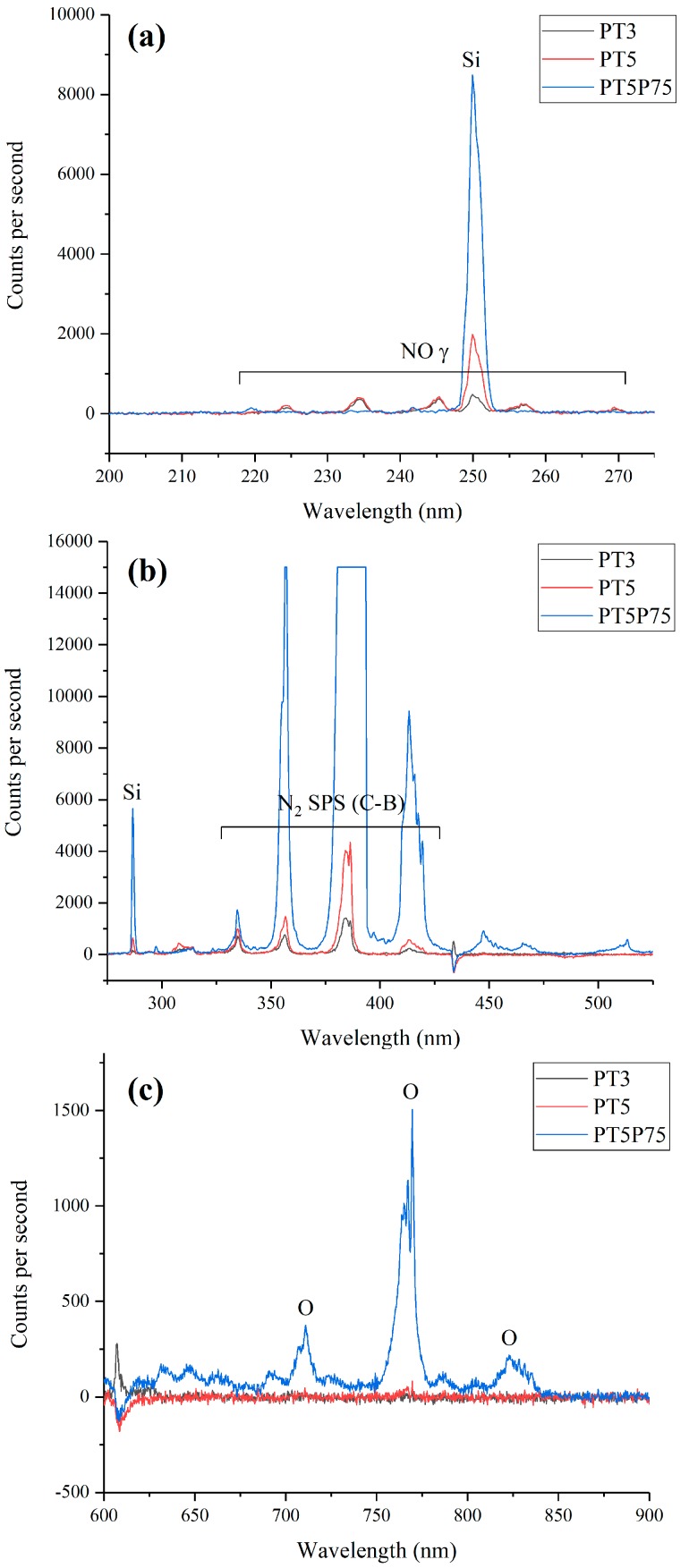
Optical emission spectra from PT3, PT5, and PT5P75 conditions. Three ranges of wavelength are chosen to highlight some of the differences. (**a**) 200–275 nm, (**b**) 275–525 nm, and (**c**) 600–900 nm.

To characterize the discharge zone, optical emission spectra were acquired from all conditions, and the results are presented in Figure 7. In all cases, spectra is dominated by the nitrogen second positive system located between 325 nm and 425 nm (Figure 7b) [41]. Signal intensity is significantly stronger in PT5P75 in all regions. This is due to the higher plasma power, which leads to a more emitting plasma (note the different scales on Y axis in Figure 7). In the near UV region (Figure 7a), NOγ system is clearly observed for PT3 and PT5 through four bands located at 234, 245, 257, and 269 nm. As the plasma power increases, the emission from NO disappeared, which may be explained by a higher presence of quenching species in the discharge zone. On the other hand, atomic O lines may be observed between 700 nm and 850 nm in the case of PT5P75 (Figure 7c), which is consistent with higher precursor fragmentation in the case of higher plasma power.

### 3.2. Surface Morphology

**Figure 8:**
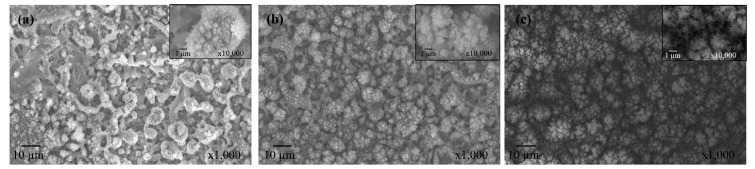
Scanning electron microscopy (SEM) images of (**a**) PT3, (**b**) PT5, and (**c**) PT5P75.

Surface morphology for different conditions was studied through scanning electron microscopy, and the results are presented in Figure 8. Comparing different surface structures, it is observed that in PT3 (Figure 8a), the precursor flow rate is not high enough for complete coverage of the pre-treated aluminum surface. On the other hand, in PT5 (Figure 8b), the substrate is fully covered by the deposition material, while the morphological features originated from the pre-treatment procedure are retained. In the case of PT5P75 (Figure 8c), micro-features from the pre-treated substrate are completely buried under the deposited material, resulting in the loss of an important roughness level. This may be due to the higher precursor fragmentation, leading to an increased presence of oxygen in the discharge zone, promoting the formation of silica powders and increasing the size of the deposited particles [48]. The presence of silica powder in the coating structure (such as the larger deposited agglomerates in Figure 8c) has an adverse effect of the mechanical stability of the coating, since larger particles become increasingly unstable and easier to remove under external forces.

### 3.3. Chemical Composition

**Figure 9:**
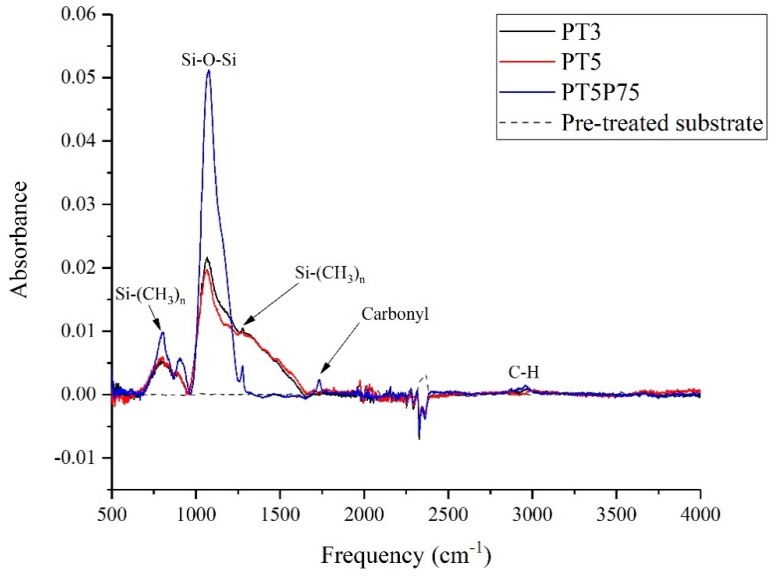
Full range of Fourier transform infrared spectroscopy (FTIR) spectra acquired from PT3, PT5, and PT5P75.

FTIR spectroscopy and XPS were used to study the chemical composition of different samples. Figure 9 shows the full range of FTIR spectra acquired from uncoated pre-treated aluminum, PT3, PT5, and PT5P75. Comparing the spectra related to the pre-treated substrate with those from the coatings, one can readily conclude that most features observed in the spectra are originated from the deposition.

In all cases, the fingerprint region of the spectrum is dominated by features common to siloxane-based coatings. The presence of bands related to Si-(CH_3_)_n_ at around 800 and 1280 cm^−1^, along with the bands related to C-H groups at around 3000 cm^−1^, confirms the deposition of organic groups through plasma polymerization. Since Si-O-Si band intensity is strongest in the case of PT5P75, the deposition thickness is significantly larger for PT5P75 compared to PT5 and PT3. The presence of carbonyl groups (C=O), evident by the sharp peak at around 1750 cm^−1^, is suggestive of higher precursor fragmentation with higher generation power. This increases the presence of oxygen in the discharge zone, which in turn enhances the formation of larger silica-based particles [48]. This is in agreement with larger deposited features observed in SEM images (Figure 8).

**Figure 10:**
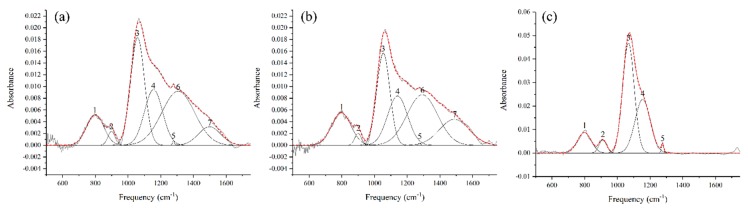
Synthetic curve models developed on the Fourier transform infrared spectroscopy (FTIR) spectra acquired for (**a**) PT3, (**b**) PT5, and (**c**) PT5P75.

In the IR spectra of siloxane-based surfaces, the 500–1700 cm^−1^ range can provide valuable information regarding the Si-O network [43]. However, since this region is populated with several organic and organosilicon-based species, the spectra should be deconvoluted into its components to reliably quantify the peak intensity and surface area. In this context, the fingerprint region of the spectra presented in Figure 9 was deconvoluted to distinguish and quantify some of the organosilicon-based species present on the surface. Figure 10 shows the results of this deconvolution and shows the locations of the synthetic curves along with their assigned vibrations.

For a brief description of what numbered components represent, see Table 3.

A notable feature of the FTIR spectra presented in Figure 10 is the continuously descending signal from 1300 cm^−1^ to 1500 cm^−1^ in the case of PT3 and PT5. Typically, this range is populated by many C-H bending vibrations in methyl (-CH_3_), methylene (=CH_2_) and methyne (=CH) [51]. However, C-H bending vibrations are usually narrower and therefore more distinct than peak indexes 6 and 7. Furthermore, this signal is not observed in the case of PT5P75, where C-H bending vibrations are also expected to occur. Therefore, it is highly unlikely that these peaks are due to C-H bending vibrations. A few other studies have observed several bands in the 1300–1500 cm^−1^ range in the case of highly porous structures with micrometric pore sizes [52,53]. These bands are not specifically identified in these studies but are only reported for highly porous surfaces. Therefore, an alternative, and more likely, explanation for this descending signal would be through the effects of surface porosity on the spectral background of the FTIR data. In PT3 and PT5, it is likely that the substrate’s pores interfere with the IR absorbance, and therefore the effects of porosity are more pronounced. In PT5P75, deposited material covers a major part of substrate porosity (see Figure 8), leading to the absence of any signal in 1300–1500 cm^−1^ range. In any case, this signal introduces a significant amount of uncertainty to any deconvolution procedure performed on this region. The main characteristic peak of Si-(CH_3_)_n_ is located at the lower boundary of this range (1275 cm^−1^), and therefore its shape and intensity is heavily affected by peak indexes 6 and 7. In this study, to avoid the uncertainty originated from the overlapping components, a different peak located at around 800 cm^−1^ (peak index 1) is used as a representative of the organic deposition. It should be noted that Peak 1 may be further deconvoluted into three separate components based on the value of n in Si-(CH_3_)_n_ [36,49,50]_._ In this study however, the resolution of the FTIR measurement is not small enough to distinguish between these components, and therefore the total surface area under this peak is used as a representative of the organic content. These various states of organic silicon will be investigated later while discussing the results of high resolution Si 2p core peak XPS spectra. Another consideration is the presence of Si-O-Si bending vibration at around 780–800 cm^−1^ [47,54], which may overlap Peak 1 in the above calculations. However, in organosilicon coatings, the intensity of Si-O-Si bending vibration is typically negligible compared to the Si-C rocking [48,55,56].

In order to determine the amount of organic functions in the Si-O-Si network, the ratio between the surface area under the Si-(CH_3_)_n_ component (peak index 1) and the surface area under quartz-like Si-O-Si component (peak index 3) is calculated. Furthermore, to determine the structural integrity of the Si-O-Si network, the ratio between the surface area under the Si-O-Si TO_2_ component (peak index 4) and the surface area under the Si-O-Si TO_1_ component (peak index 3) is calculated. Since TO_1_ is correlated with a quartz-like structure while TO_2_ is correlated with fragments of siloxane chains, a higher TO_2_/TO_1_ ratio corresponds to shorter siloxane chains and more disorder in the silica network. These ratios were calculated for PT3, PT5, and PT5P75 and are presented in Table 4, where A_n_ denotes the surface area under the n^th^ peak index.

Since the plasma power is identical for PT3 and PT5, the amount of available energy per HMDSO molecule is higher in the case of PT3. Therefore, higher monomer fragmentation is expected in lower precursor flow rates, which leads to lower Si-(CH_3_)_n_/Si-O-Si ratio. This is manifested as a lower A_1_/A_3_ ratio for PT3 than PT5. Similarly, since the precursor flow rate is identical for PT5 and PT5P75, higher power leads to higher energy per precursor molecule, which in turn increases the fragmentation and leads to lower Si-(CH_3_)_n_/Si-O-Si ratio in the case of PT5P75.

To study the structural integrity of the siloxane network, A_4_/A_3_ ratio is investigated. For PT3 and PT5, this ratio is almost identical, which is suggestive of similar siloxane structures in both cases. However, as discussed before, the main difference between PT3 and PT5 is in coating thickness and the amount of deposition (Figure 8). In the case of PT5P75, it is shown that increasing the generation power has a significant effect on the Si-O-Si network, leading to shorter siloxane chains (higher TO_2_ intensity). This is consistent with higher precursor fragmentation with higher plasma energy.

**Figure 11:**
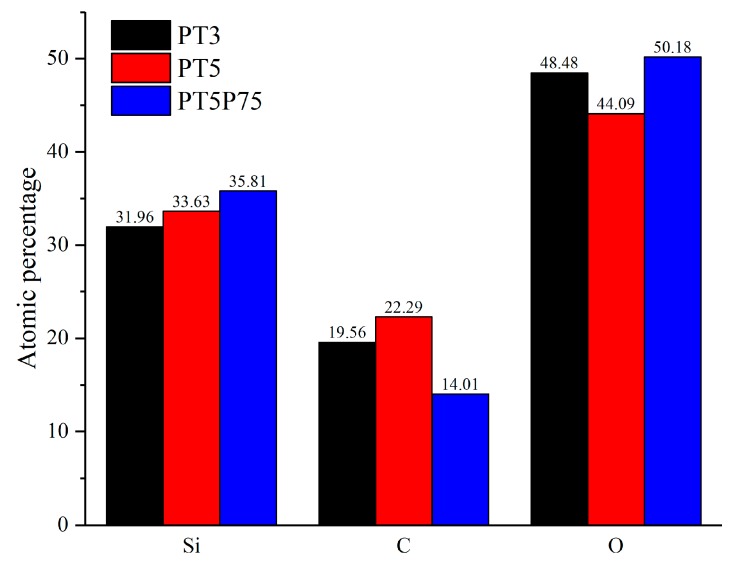
Atomic percentages of silicon, carbon and oxygen acquired from X-ray photoelectron spectrometer (XPS) for PT3, PT5, and PT5P75.

Complementary quantification of the surface chemical composition was acquired through X-ray photoelectron spectroscopy. Figure 11 shows the atomic percentages of Si, C, and O based on the survey spectra. In PT5, lower precursor fragmentation (due to the lower energy per precursor molecule) leads to the highest organic content on the surface, which is manifested as higher C percentage and lower O percentage. PT5P75 has a higher content of silicon and oxygen and a lower content of carbon, which can be linked to a decreased carbon content due to the higher precursor fragmentation. This is also in agreement with the FTIR data, where it was shown that the organic content is reduced with increasing the power. Finally, comparing PT3 with PT5, it is observed that the increased amount of energy available per precursor molecule in the case of PT3 leads to smaller amounts of deposition (lower silicon percentage) with less organic content (lower carbon percentage).

**Figure 12:**
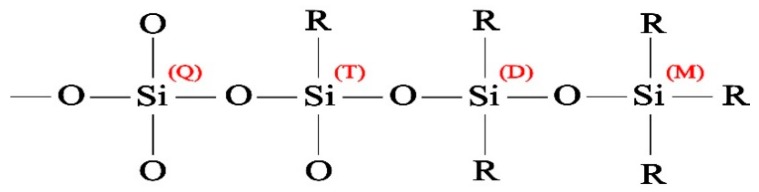
Silicon chemical states in a typical siloxane-based molecular structure.

High resolution spectra of Si 2p core peak were used to determine the chemical state of silicon atoms in the siloxane structure. Si 2p core peak analysis is done based on the method developed by O’Hare et al. [57,58] for the analysis of siloxane-based coatings using X-ray high resolution spectra of Si 2p core peak. In this method, four different chemical states of silicon atoms are identified based on the number of bonds with oxygen: Q, T, D, and M which are in contact with 4, 3, 2, and 1 oxygen atoms, respectively. These chemical states are shown schematically in Figure 12 and some further details are provided in Table 5.

**Figure 13:**
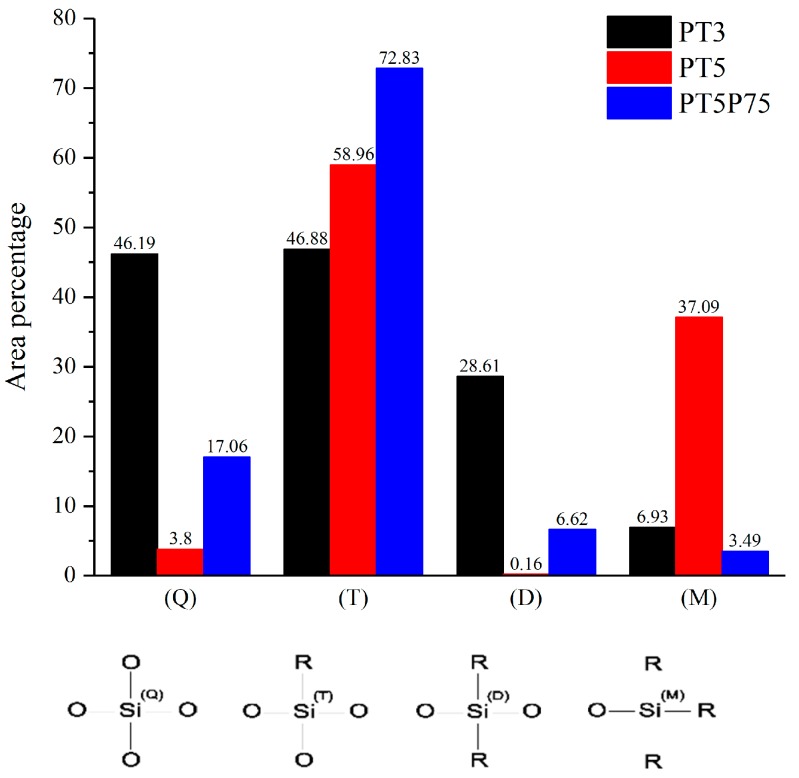
Component quantification of siloxane-based coatings determined through high resolution spectroscopy of Si 2p core peak.

Based on these chemical states and their respective binding energy in X-ray photoelectron spectra, synthetic curve-fitting models (not presented here) were developed on Si 2p core peak to determine the amount of deposited organosilicon species. These models where developed by (1) restricting the position of (Q) component to 103.69 ± 1 eV based on the calibration experiments on pure quartz samples, (2) restricting the position of (T), (D), and (M) based on their respective shifts from the position of (Q), and (3) forcing equal peak widths for all components. The results from component quantification are presented in Figure 13.

When HMDSO molecules are vaporized and injected into the flowing afterglow region of the atmospheric-pressure plasma jet, they may go through several stages of fragmentation. Si-O bonds, which are weaker than Si-C bonds, break first, generating (M) silicon states which may be deposited on the surface. Further fragmentation results in broken Si-C bonds, removing methyl groups from silicon and replacing them with oxygen atoms in the open-to-air plasma. Therefore, the presence of various silicon chemical states may be interpreted as various degrees of fragmentation. In the case presented here, higher presence of (M) groups and lower presence of (Q) groups in the topmost layer of PT5 is suggestive of lower precursor fragmentation. Even before curve-fitting, higher organic content in PT5 is evident by a 0.5 eV shift observed in Si 2p core peak position towards lower energies. This is consistent with the FTIR data, where it was shown that PT5 exhibits higher Si-(CH_3_)_n_/Si-O-Si ratio with a more organized structure. For PT3 and PT5P75, more than 90% of silicon atoms are in contact with 4 or 3 oxygen atoms (Q and T respectively), which is suggestive of high precursor fragmentation. However, in the case of PT3, (Q) and (T) are represented almost equally on the topmost layer of the coating, while in PT5P75 silicon is mostly at (T) state. Based on what has been discussed so far, it is evident that both coatings are the outcome of heavy precursor fragmentation. However, higher presence of precursor molecules in the case of PT5P75 results in the higher implementation of organic functions in the siloxane network, leading to a high concentration of (T) state in PT5P75. Similarly, lower presence of precursor molecules in PT3 reduces the functionalization of siloxane network with organic functions, leading to higher concentration of fully oxidized silicon (Q).

At this point, it should be noted that in a typical curve fitting process, the results may be examined by developing a curve model on a different peak related to a different element and confirming the agreement between the results acquired from both models. In the case presented here, this validation can potentially be done by creating synthetic models on C 1s peak based on (T), (D), and (M) functions along with other possible carbon states (such as C-C, C-H, and C=O). However, the binding energies for C 1s^(T)^, C 1s^(D)^, and C 1s^(M)^ are in a 0.5 eV range (284.7 eV, 284.5 eV, and 284.2 eV, respectively) [57]. Furthermore, the binding energy of adventitious carbon (carbon originated from exposure to air or other sources of contamination), which is typically the most prominent source of carbon in XPS data, is also in the same range. Due to the relatively large FWHM of the high-resolution spectra (~2 eV), distinguishing the peaks in such a short range is practically impossible. However, the models presented here were developed with as many physically and chemically relevant restrictions as possible (on peak position, peak width, peak shape, etc.) to ensure mathematical, physical, and chemical accuracy of component quantification. Furthermore, the results from this quantification are in complete agreement with FTIR results and the expectations based on the literature. Therefore, despite some potential limitations, we feel confident that these models can accurately represent the chemical composition of the surface.

### 3.4. Wetting Behavior

The wetting behavior of PT3, PT5, and PT5P75 is studied through static and dynamic water contact angle measurements. Numerous studies and reviews have emphasized the importance of dynamic wetting studies. It is often suggested that if a surface shows hysteresis, i.e., a difference between advancing and receding angles, static contact angle becomes an arbitrary value between advancing and receding angles [59,60,61] and tends to miss a significant amount of information regarding the wetting characteristics of a surface. In fact, it has been suggested that since the advancing angle is more sensitive to low-energy components of the surface while the receding angle is more sensitive to high-energy components [62], one can study them individually to gain a deeper insight on surface functionalities.

**Figure 14:**
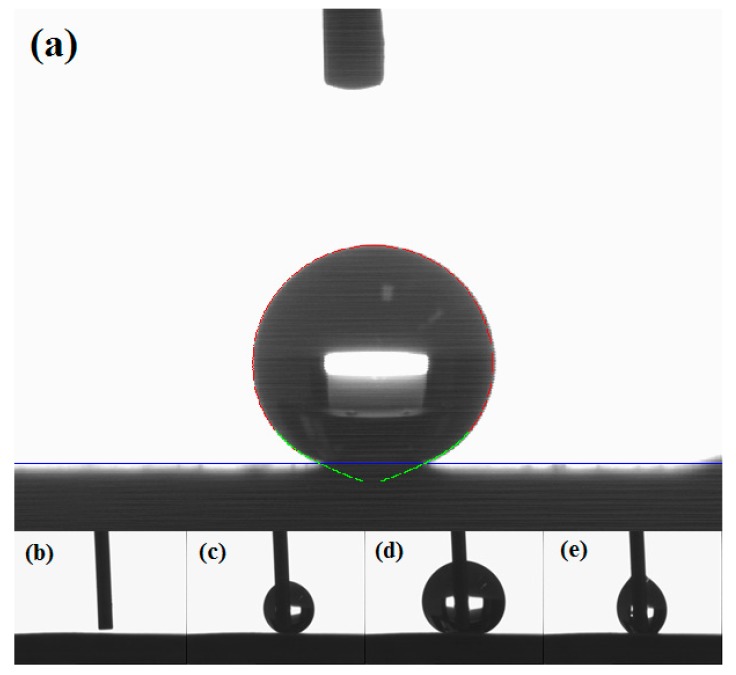
(**a**) a demonstration of the Tangent approximation method and (**b**–**e**) the procedure for determination of advancing and receding angles.

**Figure 15:**
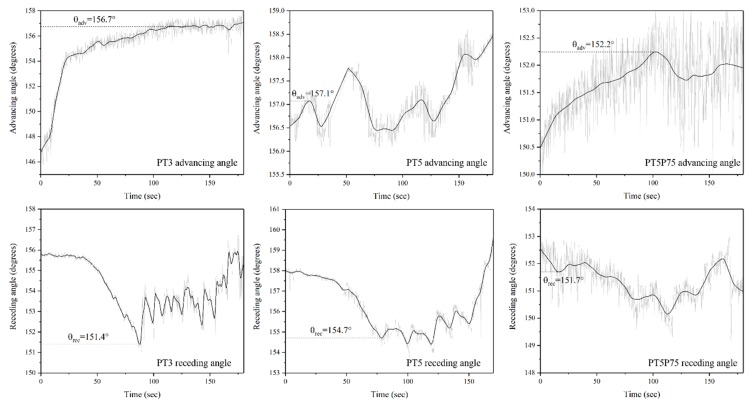
Advancing and receding angle curves for PT3, PT5 and PT5P75.

In this work, dynamic wetting is studied through placing a 4 µL droplet on the surface while keeping the needle in contact with the resting droplet and increasing or decreasing the droplet volume while measuring contact angle at the three-phase interfacial point five times per second (Figure 14). These measurements are then plotted against time and the curve is smoothed using the LOWESS method to account for minor variations (±1°) while retaining the general trend of the curves. More details on this procedure is provided in [63]. These curves are presented in Figure 15 for PT3, PT5, and PT5P75.

As the droplet volume slowly increases, the baseline initially remains the same, resulting in an increase in the measured contact angles. Eventually, the weight of the droplet becomes large enough to expand the baseline, which may be shown in the contact angle vs. time curves as either a drop in the measured values followed by a further gradual increase (sudden baseline expansion), or as a constant measured value with volume after the initial increase (continuous baseline expansion). Advancing contact angle is then determined as the measured value just before baseline expansion [39,60]. Similarly, reducing the droplet volume leads to an initial decrease with a sudden increase (sudden baseline shrinkage) or a constant value (continuous baseline shrinkage). In this case, receding angle is determined as the measured value just before the baseline shrinkage (see Figure 15). The fundamental physics behind this procedure have inspired several groups to discuss and compare their knowledge of surface chemistry, surface topography, and interfacial science [39,59,60,62,63]. However, the details of advancing and receding measurements are rarely discussed in detail in the literature, and therefore we are not certain whether other groups have observed the same behavior or their interpretation of how the baseline reacts to increasing/decreasing volume is consistent with ours.

**Figure 16:**
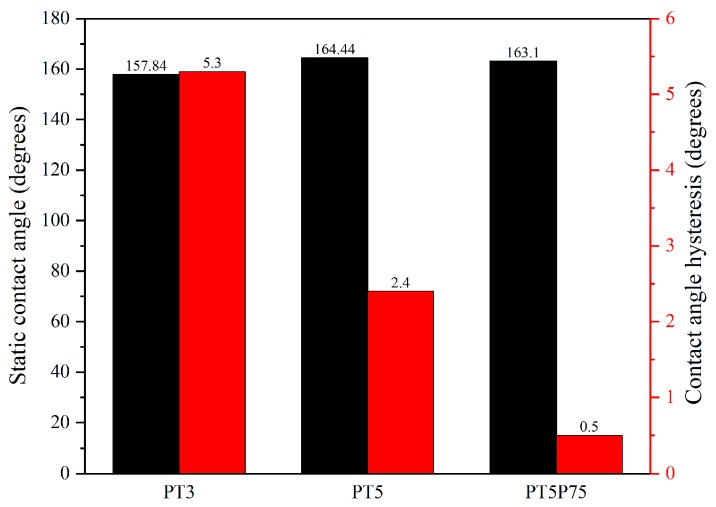
Static and dynamic contact angle for PT3, PT5, and PT5P75.

Based on the advancing and receding angle values presented in Figure 15, contact angle hysteresis was calculated for all coatings, and the results are presented in Figure 16 along with the static contact angle values. It should be noted that theoretically, the static contact angle should be between advancing and receding values. However, in the results presented here, some discrepancies may be observed due to different measurement methods: Static contact angle measurements were carried out using the Young–Laplace model, since it approximates the entire shape of the droplet, thus considering the effects of droplet weight or any other distortions in the droplet shape. However, during dynamic measurements, the needle is in contact with the droplet at all times, and therefore Young–Laplace approximation cannot identify the circular shape of the droplet. Therefore, a different approximation method (namely, the tangent method) was used, which only considers the three phase interfacial points and attempts to draw a line at this point tangent to the droplet shape to determine the contact angle.

All surfaces are shown to be superhydrophobic (WCA > 150°) with statistically insignificant variations in static contact angle due to the different deposition conditions. However, a significant difference in contact angle hysteresis (CAH) is observed for different samples, which is directly related to surface roughness. Traditionally, it is argued that hysteresis originates from defects, and thus rougher surfaces have higher CAH [59,64]. However, several studies have shown that the effects of roughness on CAH depends on the wetting regime. When water is in contact with the whole surface profile (Wenzel wetting regime), surface features act as obstacles to water motion, reducing the droplet mobility and increasing CAH. On the other hand, when roughness features are small enough so that the capillary effect prevents liquid penetration into the surface asperities (Cassie-Baxter wetting regime), water is only in contact with a small area fraction of surface features, and therefore its motion is not hindered by surface structures [62,65], increasing the droplet mobility and decreasing CAH.

In the present work, the lowest contact angle hysteresis is observed in the case of PT5P75. While presenting the chemical characteristics of the developed coatings, it was shown that precursor fragmentation in PT5P75 is higher, leading to more polar oxide content on the surface, which is expected to render the surface less hydrophobic. However, since PT5P75 exhibits higher surface roughness due to larger silica-based particles with surface features as small as only a few nanometers (see Figure 8), contact angle hysteresis is very small. In the case of PT3, surface roughness is lower due to the lower amount of deposition and large micrometric surface features originated from the pre-treatment. Finally, PT5 exhibits a CAH higher than PT3 due to the full coverage of the surface with multi-leveled structures, and lower than PT5P75 due to the smaller deposited structures.

## 4. Conclusions

In this study, superhydrophobic coatings are developed through atmospheric pressure plasma polymerization of HMDSO in the jet of nitrogen plasma produced by rotating arc discharges. The details regarding the plasma deposition process and precursor fragmentation dynamics are discussed. The plasma jet is modified by mounting a quartz tube on the jet head, thus confining the plasma jet in a smaller volume. It is shown that this modification leads to structures that are similar to what is observed in atmospheric pressure plasma polymerization of HMDSO in the absence of O_2_. After jet modification, the effects of precursor flow rate and plasma power on surface structure, wetting behavior and surface chemistry is studied. It is shown that increasing the flow rate while keeping the plasma power constant increases precursor fragmentation, leading to higher oxide deposition. On the other hand, increasing the plasma power while keeping the precursor flow rate constant results in faster deposition rates and subsequently thicker coatings, but with a higher oxide content. This demonstrates the significance of “available energy per precursor molecule” parameter, which can significantly affect the precursor fragmentation in the discharge. All conditions studied here lead to superhydrophobic surfaces with static contact angles higher than 150° and contact angle hysteresis lower than 6°.

## Figures and Tables

**Table 1:** Plasma conditions used for preparation of samples.

Sample Name	Monomer Flow Rate	Plasma Power	Plasma Duty Cycle	Jet Speed	Jet-Substrate Distance	Ionization Gas Flow Rate	Carrier Gas Flow Rate
PT3	3 g/h	500 W	50%	1 m/min	30 mm	500 L/h	400 L/h
PT5	5 g/h	500 W	50%	1 m/min	30 mm	500 L/h	400 L/h
PT5P75	5 g/h	750 W	50%	1 m/min	30 mm	500 L/h	400 L/h

**Table 2:** Acquisition parameters for survey and high-resolution X-ray photoelectron spectrometer (XPS) spectra.

Scan Type	Start Energy	End Energy	Step Width	dE	Dwell Time	# of Scans	Beam Power
Survey	1300 eV	0 eV	1 eV	4 eV	100 ms	5	150 W
High resolution	A window of ~20 eV width around the peak.		0.1 eV	0.3 eV	300 ms	10	300 W

**Table 3:** Locations and assigned chemical groups for the synthetic curves presented in Figure 10.

Peak Index	Approximate Position (cm^−1^)	Assigned to
1	800	Si-C rocking vibration in Si-(CH_3_)_n_ [36,49,50]
2	900	Si-OH bending [50]
3	1060	Si-O-Si asymmetric stretching (TO_1_)
4	1150	Si-O-Si asymmetric stretching (TO_2_)
5	1275	C-H symmetric deformation in Si-(CH_3_)_n_
6	1300–1500	
7		

**Table 4:** Ratios between surface areas under some of the components in the Fourier transform infrared spectroscopy (FTIR) curve-fitting model (Figure 10).

	A_1_/A_3_ (Si-(CH_3_)_n_/Si-O-Si)	A_4_/A_3_ (TO_2_/TO_1_)
PT3	0.35	0.67
PT5	0.52	0.64
PT5P75	0.22	0.81

**Table 5:** Chemical states of silicon in siloxane coatings [58].

	Binding Energy	Energy Shift	Function
*Q [SiO_4/2_]*	103.69 eV	0 eV	cross-linking
*T [CH_3_SiO_3/2_]*	102.89 eV	0.80 eV	cross-linking
*D [(CH_3_)_2_SiO_2/2_]*	102.21 eV	0.68 eV	propagation
*M [(CH3)_3_SiO_1/2_]*	101.85 eV	0.36 eV	termination

## References

[1] Saraf R., Lee H.J., Michielsen S., Owens J., Willis C., Stone C., Wilusz E. (2011). Comparison of three methods for generating superhydrophobic, superoleophobic nylon nonwoven surfaces. J. Mater. Sci..

[2] Ma M., Hill R.M. (2006). Superhydrophobic surfaces. Curr. Opin. Colloid Interface Sci..

[3] Guo Z., Liu W., Su B.L. (2011). Superhydrophobic surfaces: From natural to biomimetic to functional. J. Colloid Interface Sci..

[4] Johnson R.E., Dettre R.H. (1964). Contact Angle Hysteresis. III. Study of an Idealized Heterogeneous Surface. J. Phys. Chem..

[5] Guo Z., Liu W. (2007). Biomimic from the superhydrophobic plant leaves in nature: Binary structure and unitary structure. Plant Sci..

[6] Yan Y.Y., Gao N., Barthlott W. (2011). Mimicking natural superhydrophobic surfaces and grasping the wetting process: A review on recent progress in preparing superhydrophobic surfaces. Adv. Colloid Interface Sci..

[7] Li X.-M., Reinhoudt D., Crego-Calama M. (2007). What do we need for a superhydrophobic surface? A review on the recent progress in the preparation of superhydrophobic surfaces. Chem. Soc. Rev..

[8] Kale K.H., Palaskar S. (2010). Atmospheric pressure plasma polymerization of hexamethyldisiloxane for imparting water repellency to cotton fabric. Text. Res. J..

[9] Cheng Y., Zhu T., Li S., Huang J., Mao J., Yang H., Gao S., Chen Z., Lai Y. (2019). A novel strategy for fabricating robust superhydrophobic fabrics by environmentally-friendly enzyme etching. Chem. Eng. J..

[10] Favia P., D’Agostino R. (1998). Plasma treatments and plasma deposition of polymers for biomedical applications. Surf. Coat. Technol..

[11] Ellinas K., Kefallinou D., Stamatakis K., Gogolides E., Tserepi A. (2017). Is There a Threshold in the Antibacterial Action of Superhydrophobic Surfaces?. ACS Appl. Mater. Interfaces.

[12] Yao X., Song Y., Jiang L. (2011). Applications of bio-inspired special wettable surfaces. Adv. Mater..

[13] Farzaneh M. (2008). Atmospheric icing of power networks. Atmospheric Icing of Power Networks.

[14] Alizadeh A., Yamada M., Li R., Shang W., Otta S., Zhong S., Ge L., Dhinojwala A., Conway K.R., Bahadur V. (2012). Dynamics of ice nucleation on water repellent surfaces. Langmuir.

[15] Farzaneh M. (2000). Ice accretions on high–voltage conductors and insulators and related phenomena. Philos. Trans. R. Soc. Lond. A Math. Phys. Eng. Sci..

[16] Farzaneh M., Sarkar D.K. (2008). Nanostructured Superhydrophobic Coatings. J. CPRI.

[17] Marmur A. (2003). Wetting on hydrophobic rough surfaces: To be heterogeneous or not to be?. Langmuir.

[18] Li S., Page K., Sathasivam S., Heale F., He G., Lu Y., Lai Y., Chen G., Carmalt C.J., Parkin I.P. (2018). Efficiently texturing hierarchical superhydrophobic fluoride-free translucent films by AACVD with excellent durability and self-cleaning ability. J. Mater. Chem. A.

[19] Genzer J., Efimenko K. (2006). Recent developments in superhydrophobic surfaces and their relevance to marine fouling: A review. Biofouling.

[20] Ge M., Cao C., Huang J., Zhang X., Tang Y., Zhou X., Zhang K., Chen Z., Lai Y. (2018). Rational design of materials interface at nanoscale towards intelligent oil-water separation. Nanoscale Horizons.

[21] Liu H., Wang Y., Huang J., Chen Z., Chen G., Lai Y. (2018). Bioinspired Surfaces with Superamphiphobic Properties: Concepts, Synthesis, and Applications. Adv. Funct. Mater..

[22] Wang S., Feng L., Jiang L. (2006). One-step solution-immersion process for the fabrication of stable bionic superhydrophobic surfaces. Adv. Mater..

[23] Hermelin E., Petitjean J., Lacroix J.C., Chane-Ching K.I., Tanguy J., Lacaze P.C. (2008). Ultrafast electrosynthesis of high hydrophobic polypyrrole coatings on a zinc electrode: Applications to the protection against corrosion. Chem. Mater..

[24] Zhang F., Zhao L., Chen H., Xu S., Evans D.G., Duan X. (2008). Corrosion resistance of superhydrophobic layered double hydroxide films on aluminum. Angew. Chem. Int. Ed..

[25] Jafari R., Asadollahi S., Farzaneh M. (2013). Applications of plasma technology in development of superhydrophobic surfaces. Plasma Chem. Plasma Process..

[26] Martinu L., Poitras D. (2000). Plasma deposition of optical films and coatings: A review. Vac. Sci. Technol. A.

[27] Gogolides E., Constantoudis V., Kokkoris G., Kontziampasis D., Tsougeni K., Boulousis G., Vlachopoulou M., Tserepi A. (2011). Controlling roughness: From etching to nanotexturing and plasma-directed organization on organic and inorganic materials. J. Phys. D Appl. Phys..

[28] Saccaro S., Fonseca R., Veillon D.M., Cotelingam J., Nordberg M.L., Bredeson C., Glass J., Munker R. (2005). Primary plasma cell leukemia: Report of 17 new cases treated with autologous or allogeneic stem-cell transplantation and review of the literature. Am. J. Hematol..

[29] Lloyd G., Friedman G., Jafri S., Schultz G., Fridman A., Harding K. (2010). Gas plasma: Medical uses and developments in wound care. Plasma Process. Polym..

[30] Kong M.G., Kroesen G., Morfill G., Nosenko T., Shimizu T., Van Dijk J., Zimmermann J.L. (2009). Plasma medicine: An introductory review. New J. Phys..

[31] Hertwig C., Reineke K., Ehlbeck J., Knorr D., Schlüter O. (2015). Decontamination of whole black pepper using different cold atmospheric pressure plasma applications. Food Control.

[32] Pasquali F., Stratakos A.C., Koidis A., Berardinelli A., Cevoli C., Ragni L., Mancusi R., Manfreda G., Trevisani M. (2016). Atmospheric cold plasma process for vegetable leaf decontamination: A feasibility study on radicchio (red chicory, *Cichorium intybus* L.). Food Control.

[33] Merche D., Vandencasteele N., Reniers F. (2012). Atmospheric plasmas for thin film deposition: A critical review. Thin Solid Films.

[34] Siliprandi R.A., Zanini S., Grimoldi E., Fumagalli F.S., Barni R., Riccardi C. (2011). Atmospheric pressure plasma discharge for polysiloxane thin films deposition and comparison with low pressure process. Plasma Chem. Plasma Process..

[35] Foroughi Mobarakeh L., Jafari R., Farzaneh M. (2011). Superhydrophobic Surface Elaboration Using Plasma Polymerization of Hexamethyldisiloxane (HMDSO). Adv. Mater. Res..

[36] Lommatzsch U., Ihde J. (2009). Plasma polymerization of HMDSO with an atmospheric pressure plasma jet for corrosion protection of aluminum and low-adhesion surfaces. Plasma Process. Polym..

[37] Gogolides E., Ellinas K., Tserepi A. (2015). Hierarchical micro and nano structured, hydrophilic, superhydrophobic and superoleophobic surfaces incorporated in microfluidics, microarrays and lab on chip microsystems. Microelectron. Eng..

[38] Asadollahi S., Farzaneh M., Stafford L. (2018). Highly porous micro-roughened structures developed on aluminum surface using the jet of rotating arc discharges at atmospheric pressure. J. Appl. Phys..

[39] Kietzig A.M. (2011). Comments on “an essay on contact angle measurements”—An illustration of the respective influence of droplet deposition and measurement parameters. Plasma Process. Polym..

[40] Müller M., Oehr C. (2011). Comments on “an essay on contact angle measurements” by Strobel and Lyons. Plasma Process. Polym..

[41] Levasseur O., Stafford L., Gherardi N., Naudé N., Beche E., Esvan J., Blanchet P., Riedl B., Sarkissian A. (2013). Role of substrate outgassing on the formation dynamics of either hydrophilic or hydrophobic wood surfaces in atmospheric-pressure, organosilicon plasmas. Surf. Coat. Technol..

[42] Pulpytel J., Kumar V., Peng P., Micheli V., Laidani N., Arefi-Khonsari F. (2011). Deposition of organosilicon coatings by a non-equilibrium atmospheric pressure plasma jet: Design, analysis and macroscopic scaling law of the process. Plasma Process. Polym..

[43] Gunde M.K. (2000). Vibrational modes in amorphous silicon dioxide. Phys. B Condens. Matter.

[44] Kirk C.T. (1988). Quantitative analysis of the effect of disorder-induced mode coupling on infrared absorption in silica. Phys. Rev. B.

[45] Petersen J., Bardon J., Dinia A., Ruch D., Gherardi N. (2012). Organosilicon Coatings Deposited in Atmospheric Pressure Townsend Discharge for Gas Barrier Purpose: Effect of Substrate Temperature on Structure and Properties. ACS Appl. Mater. Interfaces.

[46] Launer P.J. (1987). Infrared Analysis of Organosilicon Compounds: Spectra-Structure Correlations.

[47] Barthlott W., Neinhuis C. (1997). Purity of the sacred lotus, or escape from contamination in biological surfaces. Planta.

[48] Kilicaslan A., Levasseur O., Roy-Garofano V., Profili J., Moisan M., Cote C., Sarkissian A., Stafford L. (2014). Optical emission spectroscopy of microwave-plasmas at atmospheric pressure applied to the growth of organosilicon and organotitanium nanopowders. J. Appl. Phys..

[49] Ricci M., Dorier J.L., Hollenstein C., Fayet P. (2011). Influence of argon and nitrogen admixture in HMDSO/O2 plasmas onto powder formation. Plasma Process. Polym..

[50] Park E.S., Ro H.W., Nguyen C.V., Jaffe R.L., Yoon D.Y. (2008). Infrared Spectroscopy Study of Microstructures of Poly (silsesquioxane)s. Chem. Mater..

[51] Morent R., De Geyter N., Van Vlierberghe S., Dubruel P., Leys C., Gengembre L., Schacht E., Payen E. (2009). Deposition of HMDSO-based coatings on PET substrates using an atmospheric pressure dielectric barrier discharge. Prog. Org. Coat..

[52] Coates J. (2000). Interpretation of Infrared Spectra, A Practical Approach. Encyclopedia of Analytical Chemistry.

[53] Gurav A.B., Latthe S.S., Kappenstein C., Mukherjee S.K., Rao A.V., Vhatkar R.S. (2011). Porous water repellent silica coatings on glass by sol-gel method. J. Porous Mater..

[54] Zhu Q., Chu Y., Wang Z., Chen N., Lin L., Liu F., Pan Q. (2013). Robust superhydrophobic polyurethane sponge as a highly reusable oil-absorption material. J. Mater. Chem. A.

[55] Laroche G., Fitremann J., Gherardi N. (2013). FTIR-ATR spectroscopy in thin film studies: The importance of sampling depth and deposition substrate. Appl. Surf. Sci..

[56] Raynaud P., Despax B., Segui Y., Caquineau H. (2005). FTIR plasma phase analysis of hexamethyldisiloxane discharge in microwave multipolar plasma at different electrical powers. Plasma Process. Polym..

[57] Maurau R., Boscher N.D., Guillot J., Choquet P. (2012). Nitrogen introduction in pp-HMDSO thin films deposited by atmospheric pressure dielectric barrier discharge: An XPS study. Plasma Process. Polym..

[58] O’Hare L.A., Hynes A., Alexander M.R. (2007). A methodology for curve-fitting of the XPS Si 2p core level from thin siloxane coatings. Surf. Interface Anal..

[59] O’Hare L.A., Parbhoo B., Leadley S.R. (2004). Development of a methodology for XPS curve-fitting of the Si 2p core level of siloxane materials. Surf. Interface Anal..

[60] Strobel M., Lyons C.S. (2011). An essay on contact angle measurements. Plasma Process. Polym..

[61] Montes Ruiz-Cabello F.J., Rodríguez-Valverde M.A., Cabrerizo-Vílchez M.A. (2011). Additional comments on “an essay on contact angle measurements” by M. Strobel and C. S. Lyons. Plasma Process. Polym..

[62] Di Mundo R., Palumbo F. (2011). Comments regarding “an essay on contact angle measurements”. Plasma Process. Polym..

[63] Gao L., McCarthy T.J. (2009). Wetting 101°. Langmuir.

[64] Morra M., Occhiello E., Garbassi F. (1990). Knowledge about polymer surfaces from contact angle measurements. Adv. Colloid Interface Sci..

[65] Di Mundo R., Palumbo F., D’Agostino R. (2008). Nanotexturing of polystyrene surface in fluorocarbon plasmas: From sticky to slippery superhydrophobicity. Langmuir.

